# Podocyte A20/TNFAIP3 Controls Glomerulonephritis Severity via the Regulation of Inflammatory Responses and Effects on the Cytoskeleton

**DOI:** 10.3390/cells14050381

**Published:** 2025-03-05

**Authors:** Paulina Köhler, Andrea Ribeiro, Mohsen Honarpisheh, Ekaterina von Rauchhaupt, Georg Lorenz, Chenyu Li, Lucas Martin, Stefanie Steiger, Maja Lindenmeyer, Christoph Schmaderer, Hans-Joachim Anders, Dana Thomasova, Maciej Lech

**Affiliations:** 1 Renal Division, Department of Medicine IV, Ludwig-Maximilians-University (LMU) Hospital, Ludwig-Maximilians-University (LMU), 80336 Munich, Germany; 2 Klinikum Rechts der Isar, Department of Nephrology, Technical University Munich (TUM), 80333 München, Germany; 3 III. Department of Medicine, University Medical Center Hamburg-Eppendorf, 20251 Hamburg, Germany; 4 Institute of Biology and Medical Genetics, 2nd Faculty of Medicine, Charles University Prague and University Hospital Motol, 15006 Prague, Czech Republic

**Keywords:** A20, Tnfaip3, podocyte, inflammation, anoikis

## Abstract

A20/Tnfaip3, an early NF-κB response gene and key negative regulator of NF-κB signaling, suppresses proinflammatory responses. Its ubiquitinase and deubiquitinase activities mediate proteasomal degradation within the NF-κB pathway. This study investigated the involvement of A20 signaling alterations in podocytes in the development of kidney injury. The phenotypes of A20Δpodocyte (podocyte-specific knockout of A20) mice were compared with those of control mice at 6 months of age to identify spontaneous changes in kidney function. A20Δpodocyte mice presented elevated serum urea nitrogen and creatinine levels, along with increased accumulation of inflammatory cells—neutrophils and macrophages—within the glomeruli. Additionally, A20Δpodocyte mice displayed significant podocyte loss. Ultrastructural analysis of A20 podocyte-knockout mouse glomeruli revealed hypocellularity of the glomerular tuft, expansion of the extracellular matrix, podocytopenia associated with foot process effacement, karyopyknosis, micronuclei, and podocyte detachment. In addition to podocyte death, we also observed damage to intracapillary endothelial cells with vacuolation of the cytoplasm and condensation of nuclear chromatin. A20 expression downregulation and CRISPR-Cas9 genome editing targeting A20 in a podocyte cell line confirmed these findings in vitro, highlighting the significant contribution of A20 activity in podocytes to glomerular injury pathogenesis. Finally, we analyzed *TNFAIP3* transcription levels alongside genes involved in apoptosis, anoikis, NF-κB regulation, and cell attachment in glomerular and tubular compartments of kidney biopsies of patients with various renal diseases.

## 1. Introduction

Numerous kidney diseases exhibit progressive glomerular inflammation, and diverse regulatory mechanisms exist to control unwanted inflammatory signals. Dysregulation of these mechanisms often results in significant tissue injury and loss of function [1].

While uncontrolled inflammatory responses affect all cell types in damaged tissue, cellular reactions can vary notably. For example, immune cells such as dendritic cells possess receptor repertoires and regulatory mechanisms distinct from epithelial cells and their need for a measured response to pathogen-associated molecular patterns owing to consistent exposure to the environment and commensal flora. Podocytes, highly specialized kidney cells within the glomeruli, are crucial for the glomerular filtration barrier and operate under constant stress [2]. Sustaining podocyte homeostasis is essential for preserving cellular integrity and proper metabolism. Furthermore, maintaining podocyte homeostasis is vital for optimal glomerular filtration and overall glomerular health. The development of glomerular inflammation may lead to progressive filtration barrier malfunction with podocyte effacement, proteinuria, and eventual renal failure [3]. Podocytes exert a notable influence on inflammation themselves, given their expression of diverse pattern recognition receptors (PRRs) [4,5]. For example, they express functional Toll-like Receptor (TLR3) and RIG-I-like Helicase (RLH) signaling pathways [6], TLR2/4 [7,8,9], other TLRs [10,11], Nucleotide-binding Oligomerization Domain-containing proteins (NODs) [12], C-type Lectin Receptors (CLRs) [13,14], and inflammasome molecules [15,16]. PRR signaling is rapid, commences within minutes, and necessitates precise pathway regulation that is universally applicable across cell types to prevent weeks-long chronic inflammation with possible tissue damage. Moreover, podocytes further resemble inflammatory cells through the expression of Major Histocompatibility Complex class II (MHCII) [17], a costimulatory molecule for T cell activation in B7-1 [3] and Neonatal Fc Receptor (FcRn) [18], which enables them to operate as antigen-presenting cells (APCs) that activate T cells [19]. These immune functions necessitate robust endogenous checkpoints to ensure that inflammatory responses are both effective and tightly regulated, preventing excessive or prolonged activation. One such vital regulatory checkpoint in inflammatory signaling is the ubiquitin-modifying enzyme A20, also known as Tumor Necrosis Factor Alpha-Induced Protein 3 (TNFAIP3). Deficiency or dysregulation of A20 has proven to be pivotal in the emergence of several inflammatory disorders, including rheumatoid arthritis [20], inflammatory bowel disease [21], and Crohn’s disease [22]. Its anti-inflammatory function, particularly within the immune responses of myeloid cells, has been extensively characterized in several relevant studies, underscoring its pivotal role in maintaining immune homeostasis. The consequences of A20 deficiency and its impact on inflammation have also been shown in various other innate and adaptive immune cells [23]. Initially, renowned for its capacity to inhibit Nuclear Factor kappa-light-chain-enhancer of activated B cells (NF-κB) signaling and Tumor Necrosis Factor Alpha (TNF-α) responses, A20 has since been shown to exhibit a robust influence on cell death processes. Its inherent expression curbs TNFα-induced cell apoptosis, thereby providing an additional layer of protection against inflammatory-induced cellular damage.

Interestingly, A20-dependent inhibition of NF-κB has been shown to promote necrotic cell death, a function that contrasts with its antiapoptotic role in cytokine-induced apoptosis [24]. According to the authors of this study, A20-mediated NF-κB inhibition delineates cellular sensitivity to Reactive Oxygen Species (ROS)-induced death. Cells with reduced A20 expression exhibit enhanced resistance to necrotic cell death triggered by oxidative stress [24]. This study aimed to elucidate the role of A20, a negative regulator of inflammation in podocytes, as its function within these cells remains uncharted. Given their indispensable role within the glomerulus, unique physiology, and limited proliferative capacity, podocytes provide crucial insights into the complex pathogenesis of various renal diseases. These investigations underscore that A20 deserves further classification as a pivotal protein for preserving glomerular homeostasis under normal conditions, as well as a potent controller of inflammatory progression in response to injury.

## 2. Materials and Methods

Animal experiments: A20-flox/flox-Nphs2-cre+ (A20PKO, A20Δpodocyte) mice were generated by breeding A20-flox/flox mice, in which loxP sites flanked exons 4 and 5 of the A20 gene, with Nphs2-cre+ mice expressing Cre recombinase under the control of the podocin promoter. This conditional A20 allele allows the tissue/cell-specific inactivation of A20 (out-of-frame transcript) through the expression of Cre recombinase. Both mouse strains were backcrossed on a C57/B6 background, and both strains have been previously described [25,26]. The experimental group consisted of A20-flox/flox-Nphs2-cre+ mice, while the control (Co.) group included mice with either single flox or cre mutations. No differences were observed between the flox+ and cre+ control groups. Additionally, the single mutation had no detectable effect on the phenotype of the mice or on the podocyte phenotype. Spontaneous glomerular damage was characterized in 24-week-old female mice (*n* = 8–10 per group). We used female mice to avoid enhanced hierarchical behavior during long-term cage housing. Such behavior could lead to injuries that might interfere with the experiments or necessitate premature termination, compromising the study’s integrity. We did not observe any differences between male and female mice in our preliminary studies. The animal experiments were performed in accordance with the European law regarding the protection of animal welfare and with the approval of the local government authorities, Regierung von Oberbayern (reference number: 55.2-1-54-2532-63-12 from 27 February 2015).

Histological analysis and renal function parameters: Kidney tissues were fixed in 4% paraformaldehyde and embedded in paraffin. The 2–4 µm sections were subjected to periodic acid–Schiff (PAS) staining for Wilms’ tumor 1 (WT-1) and immunostaining. For glomerular immune cell infiltration and WT-1- staining, a number of immune cells and WT-1+ cells were evaluated. For each kidney, at least 10 glomeruli were analyzed. Leukocytes were detected by immunostaining with a rat anti-mouse Ly-6B.2 antibody (Serotec, UK), macrophages were detected via Macrophage Antigen 2 (Mac2) staining (Serotec, Oxford, UK, 1:50), and T lymphocytes were visualized via a rat anti-mouse CD3 antibody (Serotec, UK). Histological analyses were conducted in a blinded manner, with the scientists unaware of which samples belonged to the experimental group and which to the control group. Serum creatinine levels were measured via the Jaffé method, and blood urea nitrogen (BUN) was measured via an enzymatic test according to the manufacturer’s instructions (Diasys, Holzheim, Germany). The presence of protein in the urine samples was tested. Urinary creatinine was determined via the standard Jaffé reaction. The urinary protein–creatinine ratio (UPCR) in random urine samples was used as an indicator of significant proteinuria (to avoid 24 h urine collection in metabolic cages) [27].

RNA extraction, reverse transcription, and qRT–PCR: A Norgen total RNA Kit (Norgen Biotek, Thorold, ON, Canada) was used to extract total RNA from renal tissue stored in RNAlater, according to the manufacturer’s instructions. cDNA was synthesized from 1 µg of total RNA via reverse transcription polymerase chain reaction (PCR) via Superscript II reverse transcriptase (Thermo Fisher, Berlin, Germany) according to the manufacturer’s instructions. Quantitative real-time PCR (qRT–PCR) of cDNA was performed with a Light Cycler 480 (Roche, Munich, Germany). *18S* rRNA or Glyceraldehyde 3-Phosphate Dehydrogenase (*GAPDH*) mRNA was used as a reference transcript for relative quantification. Controls consisting of ddH2O were negative for the target and reference genes. The melting curve profiles were analyzed. Amplicons were visualized on agarose gels to evaluate unspecific products. All primers used for amplification (Supplementary Table S1) were purchased from Metabion (Martinsried, Germany). qPCR was performed after 3 or 18 h of stimulation with LPS. The time points were chosen based on the kinetics of transcript changes: the shorter 3-h time point was suitable for proliferation-related genes, chemokines, and cytokines, while the 18-h time point was selected to focus on cytoskeletal changes.

In vitro cell experiments: The murine immortalized podocytes, K5P5, and A20-deficient murine-immortalized podocytes, K5P5^A20−/−^, were cultured as described [28]. In brief, the cells were grown on collagen A until confluent on coated plates in 33C in RPMI supplemented with 10% Fetal Calf Serum (FCS), 1% Penicillin–Streptomycin (PS), and 100 ng/mL mouse recombinant Interferon Gamma (IFN-γ) (Immunotools). For differentiation and experiments, 100,000 cells were plated on 12-well plates or 300,000 cells were plated on 6-well plates in 0.5 mL or 1 mL medium without IFN-γ, respectively. The cells were used on day 10. For the siRNA experiments, a “reverse transfection” protocol was used. Transfection complexes (120 nM siRNA + 4 µL/mL GeneMute in FCS/PS-free medium) were incubated for 15 min and mixed with the freshly passaged differentiated cells and plated as indicated above. The medium was changed after 6 h to complete 10% FCS RPMI medium. The cells were stimulated 24 h after transfection with 100 ng/mL ultrapure LPS (InvivoGen, Toulouse, France) for 3 h. For testing cytotoxicity and cell death, we used an in vitro lactate dehydrogenase (LDH) assay (Roche, Munich, Germany), which was performed according to the manufacturer’s instructions. Briefly, 10,000 cells were seeded per well in a 96-well plate in complete media and incubated overnight. The medium was changed to media supplemented with 2% FCS. The cells were stimulated with 100 ng/mL LPS and 100 ng/mL TNFa for 24 h. The supernatant was used to measure lactate dehydrogenase at 492 nm. Triton X 1% served as a positive control, and unstimulated cells and media served as negative controls. Cell viability and metabolic activity were assessed via the MTT (3-(4,5-dimethylthiazol-2-yl)-2,5-diphenyltetrazolium bromide) assay according to the manufacturer’s instructions. Briefly, 4 × 10^4^ cells were seeded per well in a 96-well plate in complete media overnight. The medium was then changed with 0% FCS as a negative control and 2%, 10%, and 20% as positive controls for 72 h. MTT was added to each well, and the mixture was incubated for 2 h. The reaction was stopped, and the absorbance was measured at 570 nm. For Annexin V and Propidium Iodide (PI) staining, cells were stained using a BD kit following the manufacturer’s protocol. Briefly, cells were incubated with fluorochrome-conjugated Annexin V to detect early apoptosis, and with PI to identify necrotic or late apoptotic cells. Flow cytometry was performed to analyze the staining pattern, distinguishing viable, early apoptotic, and necrotic cells. The cells were stimulated with 100 ng/mL ultrapure LPS (InvivoGen) for 6 h. Bone marrow was isolated from the tibiae of mice. CD11b+ and CD11c+ cells were isolated from the spleen using magnetic beads following standard protocols (Miltenyi Biotec, Gladbach, Germany). Neutrophils were purified from blood using bead-based isolation according to standard procedures (Miltenyi Biotec Gladbach, Germany). Kidney tubules and glomeruli were micro-dissected, and the material obtained was sufficient for quantitative PCR. For human studies, prenormalized cDNAs derived from poly(A)-selected DNase-treated RNAs, purified from pools of healthy human tissues, were obtained from Clontech (Mountain View, CA, USA).

Plasmids and CRISPR/Cas9-based gene KO: To knock out the Tnfaip3 gene in murine podocyte cells, guide RNAs (gRNAs) were designed using the CRISPR Design Tool on the Benchling database, following a previously described protocol [29]. Four gRNAs targeting exon 1 were selected based on their on-target efficiency and minimal off-target effects. Single-stranded gRNAs (Metabion, Martinsried, Germany) were converted to double-stranded gRNAs by incubation with T4 PNK enzyme (New England BioLabs, Ipswich, MA, USA) [30]. The resulting double-strand oligo DNA for each gRNA was cloned into the BbsI site of the SpCas9-2A-GFP plasmid PX458 (Addgene, Cambridge, MA, USA), enabling simultaneous expression of sgRNA and Cas9. Digestion and ligation reactions were conducted using FastDigest BbsI (Fermentas), T4 ligase (New England BioLabs), Dithiothreitol (DTT), Adenosine Triphosphate (ATP), and Tango buffer (Fermentas, Vilnius, Lithuania), with incubation cycles alternating between 37 °C and 21 °C for 1 h. Residual linear DNA was digested using PlasmidSafe exonuclease, and the processed plasmids were transformed, purified, and sequenced to confirm the gRNA inserts. Murine podocytes were transfected with the expression plasmids using Lipofectamine 2000 (Thermo Fisher Scientific, Waltham, MA, USA), following the manufacturer’s instructions. After 24 h, GFP-positive cells were isolated by flow cytometry, sorted as single cells, and seeded into 96-well plates. These single-cell clones were maintained for two weeks to allow clonal expansion.

Immunofluorescence: Phalloidin staining: Podocytes were cultured on cover slips placed in a 6-well plate, followed by LPS or vehicle treatment for 24 h. The cells were fixed for 15 min in 4% formaldehyde and then washed three times in PBS. The cells were permeabilized in PBS containing 0.1% Triton X-100 for 5 min and washed 3 times with PBS. The prepared cells were stained with a 1:20 dilution of Alexa Fluor^®^ 488-phalloidin (Sigma, Darmstadt, Germany) for 40 min at room temperature in the dark. The stained cells were washed three times in PBS. After the cover slips were mounted with DAPI and the cells were stained onto glass slides, the stained cells were examined and imaged under a fluorescence microscope. For SYTOX staining, 1 × 10^4^ cells were seeded on 8-well slides (Ibidi, Gräfelfing, Germany) and stimulated with 100 ng/mL LPS or vehicle for 24 h. SYTOX dye (Thermo Fisher Scientific) was used to stain dead cells. Briefly, the cells were washed two times with PBS, and 5µM SYTOX dye was added to the cells for 15 min. The cells were washed two times with PBS to remove excess dye and subjected to fluorescence microscopy.

Patients and Microarray Analysis: Human renal biopsy specimens and associated Affymetrix microarray expression data were obtained through the European Renal cDNA Bank-Kröner-Fresenius Biopsy Bank initiative [31]. All biopsies were collected with patients’ informed consent and approval from respective local ethics committees. Post biopsy, the tissue was transferred to RNase inhibitor and micro-dissected into glomeruli and tubulointerstitium. Total RNA was extracted, reverse-transcribed, and amplified [32]. The processes of fragmentation, hybridization, staining, and imaging followed the Affymetrix Expression Analysis Technical Manual (Affymetrix, Santa Clara, CA, USA). In this study, we used published gene expression profiles (Affymetrix GeneChip Human Genome U133A and U133 Plus2.0 Arrays; GSE99340, GSE32591, GSE35489, GSE37463, GSE47185) for analysis. CEL file normalization was carried out using the Robust Multichip Average method via RMAExpress (Version 1.0.5) with the human Entrez-Gene custom CDF annotation from Brain Array version 25 (http://brainarray.mbni.med.umich.edu/Brainarray/Database/CustomCDF/CDF_download.asp accessed on 5 January 2021). Batch effect corrections were implemented using ComBat from the GenePattern pipeline (http://www.broadinstitute.org/cancer/software/genepattern/ accessed on 5 January 2021). Differentially expressed genes were identified using the Significance Analysis of Microarrays (SAM) method through TiGR (MeV, Version 4.8.1) [33]. Data from patients diagnosed with Hypertensive Nephropathy/HT(N) (Glom *n* = 15; Tub: *n* = 21), Minimal Change Disease/MCD (Glom *n* = 14; Tub: *n* = 15), IgA Nephritis/IgA (Glom *n* = 27; Tub: *n* = 26), Rapidly Progressive Glomerulonephritis/RPGN (Glom *n* = 15; Tub: *n* = 21), Systemic Lupus Erythematosus/SLE (Glom *n* = 32; Tub: *n* = 32), Membranous Glomerulonephritis/MGN (Glom *n* = 21; Tub: *n* = 18), Focal Segmental Glomerulosclerosis/FSGS (Glom *n* = 23; Tub: *n* = 13), and Diabetic Nephropathy/DN (Glom: *n* = 14; Tub: *n* = 18) were included for differential gene expression analysis. Kidney biopsies from living donors prior to transplantation (LD, Glom: *n* = 41; Tub: *n* = 42) served as controls.

Statistical analysis: The data are expressed as the means ± SEMs. Data from control and A20-deficient mice were compared with ANOVA on ranks, followed by the Student–Newman–Keuls test for comparing three or more sample means via GraphPad Prism Software 5. Student’s *t* test was used for direct comparisons between control and A20-deficient cells/mice in the case of normally distributed data or sample sizes of *n* > 15. The Mann–Whitney U test was used to analyze data with a small sample size and nonparametric distribution. A *p* value < 0.05 indicated statistical significance.

## 3. Results

### 3.1. Characterization of A20 Expression in Mouse Podocytes

**Figure 1:**
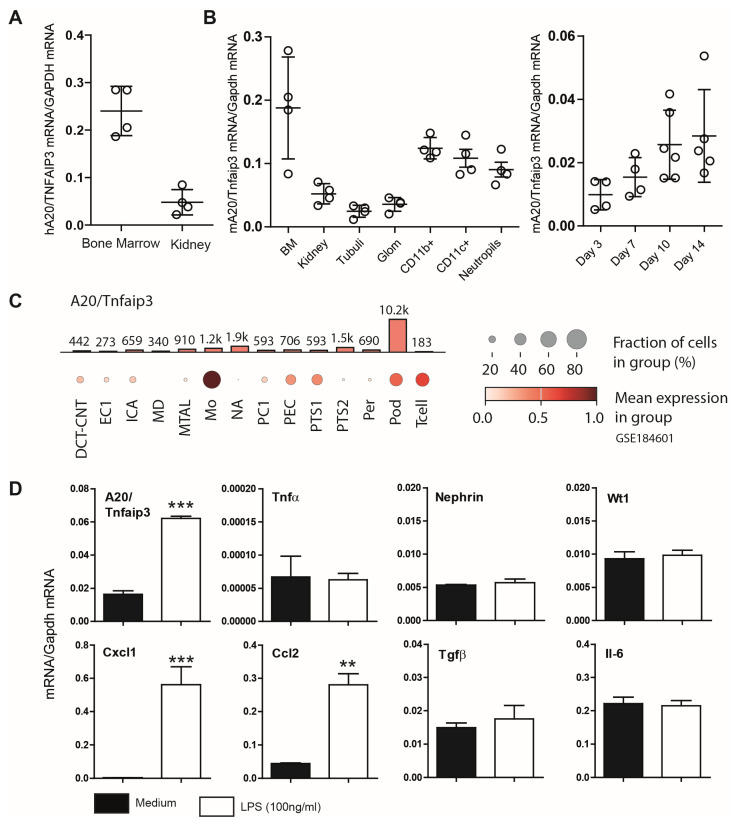
(**A**) Basal mRNA expression of *A20/Tnfaip3* in human tissues. Quantitative real-time PCR analysis of prenormalized cDNA derived from poly-(A)-selected DNase-treated RNA isolated from human bone marrow and kidney. Transcript expression levels were calculated via the use of human glyceraldehyde-3-phosphate dehydrogenase (*GAPDH*) as a housekeeping gene. The data are shown as the means ± SDs. (**B**) Quantitative real-time PCR analysis of cDNA derived from RNA isolated from murine (C57BL/6) tissues and kidney cells/compartments as well as K5P5 cells after day 3, 7, 10, and 14 of differentiation was performed as described in the Section 2. The detected mRNA expression levels were calculated via the use of murine *Gapdh* as a housekeeping gene. Data are shown as the means ± SDs. (**C**) The data show the expression pattern of *Tnfaip3* in the kidney (GSE184601). The analysis indicates high *Tnfaip3* expression in monocytes (Mos), glomeruli parietal epithelial cells (PECs), proximal tubule segment 1 (PTS1), podocytes (Pods), and T lymphocytes (Tcell). The analysis indicates low *Tnfaip3* expression in intercalated cell type A (ICA), distal convoluted tubule–connecting tubule (DCT-CNT), intercalated cell type A (ICA), macula densa (MD), thick ascending limb of henle in medulla (MTAL), principal cell 1 (PC1), proximal tubule segment 2 (PTS2), and pericyte (Per). (**D**) A20 mRNA induction following 3 h of LPS (100 ng/mL) stimulation in cultured podocytes. Total RNA was then collected to quantify gene expression via RT–PCR. The data are shown as the means ± SEMs and represent one of two independent experiments (*n* = 3); ** *p* < 0.01 and *** *p* < 0.001.

We observed A20 expression in immune cells as well as renal tissues, including both glomerular and tubular compartments (Figure 1A,B). A20 expression remained stable in differentiated podocytes and was detectable from day 3 to day 14 (Figure 1B). RNA-seq data from murine FACS-sorted podocytes showed *Tnfaip3* expression at 157 fragments per kilobase million [34] and additional sequencing data showed significant abundance of *Tnfaip3* in podocytes compared to other renal cells (Figure 1C) [35]. These findings prompted us to investigate the role of A20 in podocyte injuries. First, we observed that the *Tnfaip3* transcript encoding A20 protein is significantly upregulated following stimulation with lipopolysaccharide (LPS) (Figure 1D). Interestingly, while LPS stimulation altered the expression of chemokines such as C-X-C motif chemokine ligand 1 (*Cxcl1*) and C-C motif chemokine ligand 2 (*Ccl2*), the expression of *Tnf-α* remained unchanged after 24 h (Figure 1D).

**Figure 2:**
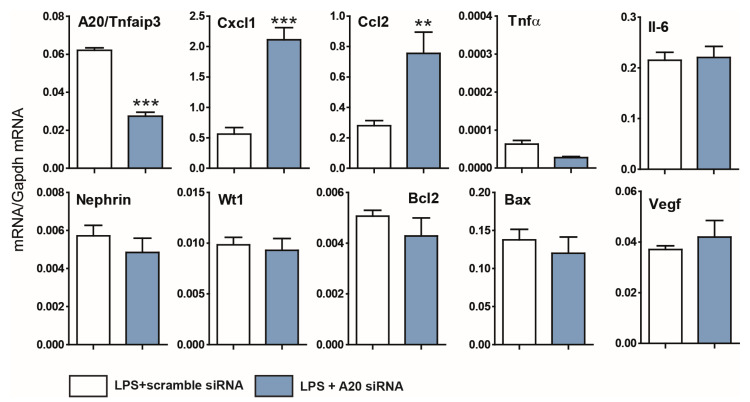
Effects of A20 knockdown and activation on proinflammatory and profibrotic factors in podocytes. A20 knockdown or control (scrambled) cells were incubated for 3 h in the presence of 100 ng/mL LPS. Total RNA was then collected to quantify gene expression via RT–PCR. The data are shown as the means ± SEMs and represent one of two independent experiments (*n* = 3); ** *p* < 0.01 and *** *p* < 0.001.

Consequently, our primary objective was to explore the potential role of A20 in podocyte injuries. First, we used an siRNA experimental approach to deplete *Tnfaip3* transcripts in the K5P5 podocyte cell line. Transfection with siRNA targeting *Tnfaip3* resulted in a marked reduction of over 50% in A20-encoding transcript (Figure 2). To assess the effects of A20 knockdown on the inflammatory response and cell viability in the context of inflammation, the cells were exposed to LPS for 3 h. To mitigate potential discrepancies in cell count and viability, expression levels were normalized against the cell number, total RNA content, and housekeeping genes. Depletion of the *Tnfaip3* gene led to a substantial increase in *Cxcl1* and *Ccl2* chemokine secretion, with no significant alterations observed in *Tnf-α* (a gene with inherently low expression in podocytes) or the podocyte-specific markers *nephrin* and *Wt1* (Figure 2).

In summary, A20 represses chemokine expression in podocytes. The shifts observed in the pro-chemotactic effects following A20 depletion motivated our pursuit of a comprehensive *Tnfaip3* gene deletion investigation.

### 3.2. A20Δpodocyte Mice Exhibit Enhanced Glomerular Inflammation

**Figure 3:**
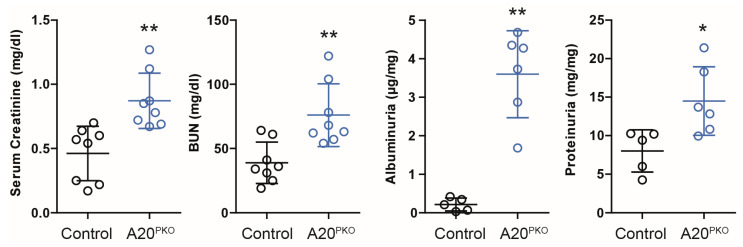
Phenotyping of A20Δpodocyte mice at the age of 6 months reveals differences in serum creatinine, BUN, and proteinuria. A20Δpodocyte mice were compared with Cre- or Flox- littermate controls. The data are shown as the means ± SDs. * *p* < 0.05, ** *p* < 0.01.

We initially assessed whether specific A20 deficiency in podocytes is associated with spontaneous glomerular changes in 24-week-old A20Δpodocyte mice compared with control mice. A20Δpodocyte mice were generated as described in the Section 2. As anticipated, we noted statistically significant differences in the serum creatinine and BUN levels between the A20Δpodocyte and control mice (Figure 3). Furthermore, A20Δpodocyte mice exhibited accelerated albuminuria and proteinuria compared to control mice.

**Figure 4:**
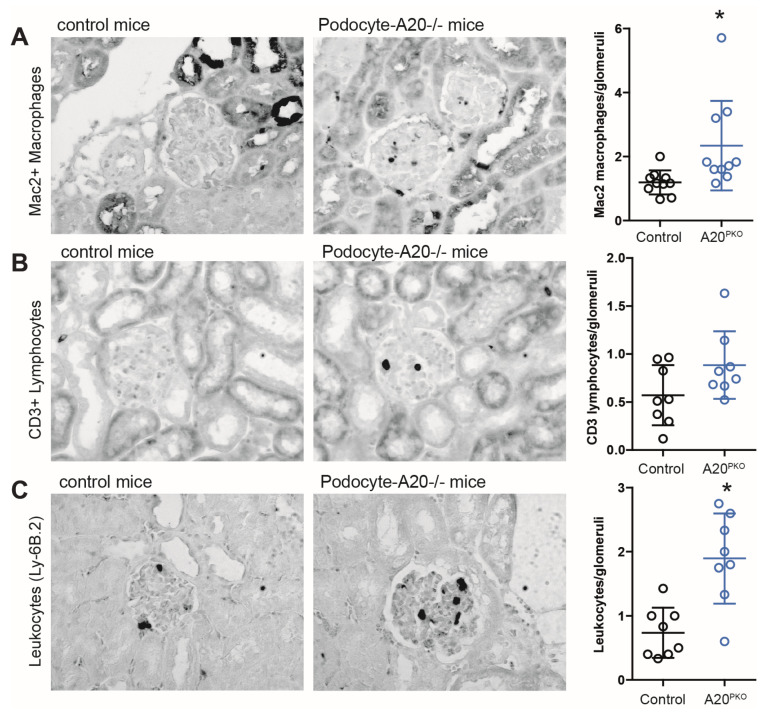
Phenotyping of A20Δpodocyte mice at the age of 6 months reveals differences in the infiltration of immune cells in glomerulus. Sections of kidneys from control and A20Δpodocyte mice were stained for glomerular macrophage (**A**), CD3+ lymphocyte, (**B**) and leukocyte (**C**) infiltration at the age of 6 months. The number of infiltrating cells was assessed in at least 15 glomeruli per kidney (*n* = 8–10 animals per group). The data are shown as the means ± SDs. * *p* < 0.05.

Next, we investigated whether a decrease in A20 expression attenuated kidney changes in mice. Histologic evaluation of glomeruli revealed glomerulonephritis, characterized by mesangial matrix expansion and thickening of peripheral capillary walls. However, no discernible differences were noted in the interstitial compartment in the kidney between the phenotypes. To elucidate the glomerular inflammatory processes regulated by A20, we analyzed glomerular immune cell infiltration and investigated the expression of inflammatory genes. A20Δpodocyte mice presented significantly greater numbers of glomerular Mac2+ macrophages and leukocytes, whereas CD3+ cell counts remained unchanged (Figure 4).

**Figure 5:**
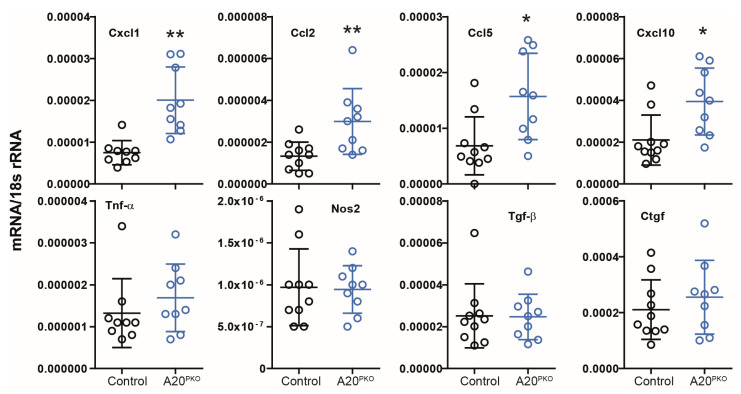
Phenotyping of A20Δpodocyte mice at the age of 6 months reveals differences in the relative mRNA expression of the indicated genes in the renal cortex. The data are shown as the means ± SDs. * *p* < 0.05 and ** *p* < 0.01.

We subsequently investigated gene expression in the kidney cortex. Real-time quantitative PCR analysis revealed transcripts of adhesion molecules, profibrotic factors, chemokines, and cytokines. As shown in Figure 5, mRNA expression levels of several chemokines, such as *Cxcl1*, *Cxcl10*, *Ccl2*, and *Ccl5*, were elevated in A20Δpodocyte mice. Consistent with our observations in A20 knockdown podocytes, we did not observe marked differences in *Tnf-α* expression. Moreover, the kidneys of A20Δpodocyte mice did not exhibit elevated mRNA levels of transforming growth factor-beta (*Tgf-β*) or connective tissue growth factor (*Ctgf*) compared with those in control kidneys.

In summary, these findings indicate an increased basal level of inflammation in the glomeruli of podocyte-specific A20-deficient mice. These results suggest that A20 plays a critical role in podocytes by regulating chemokines necessary for leukocyte recruitment and providing protection from proteinuria.

### 3.3. A20Δpodocyte Mice Exhibit Increased Glomerular Injury

**Figure 6:**
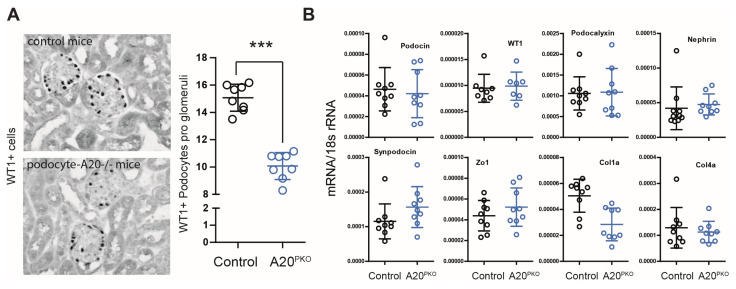
(**A**) Representative pictures showing glomerular WT1 staining in control and A20Δpodocyte mice at the age of 6 months. The WT1-positive cells in the groups were quantified by counting WT1+ cells in 15 glomerular sections per kidney. The data are shown as the means ± SDs. *** *p* < 0.001 versus controls. (**B**) Relative mRNA expression of the indicated genes in the renal cortex of control and A20Δpodocyte mice.

Next, we quantified the number of podocytes by counting WT1-positive cells within the glomeruli. Compared with control mice, A20Δpodocyte mice presented a lower podocyte count at 24 weeks of age (Figure 6A). However, compared with control mice, A20Δpodocyte mice did not exhibit reduced levels of transcript encoding podocin, nephrin, Wilms’ tumor 1, or synpodocin (Figure 6B).

**Figure 7:**
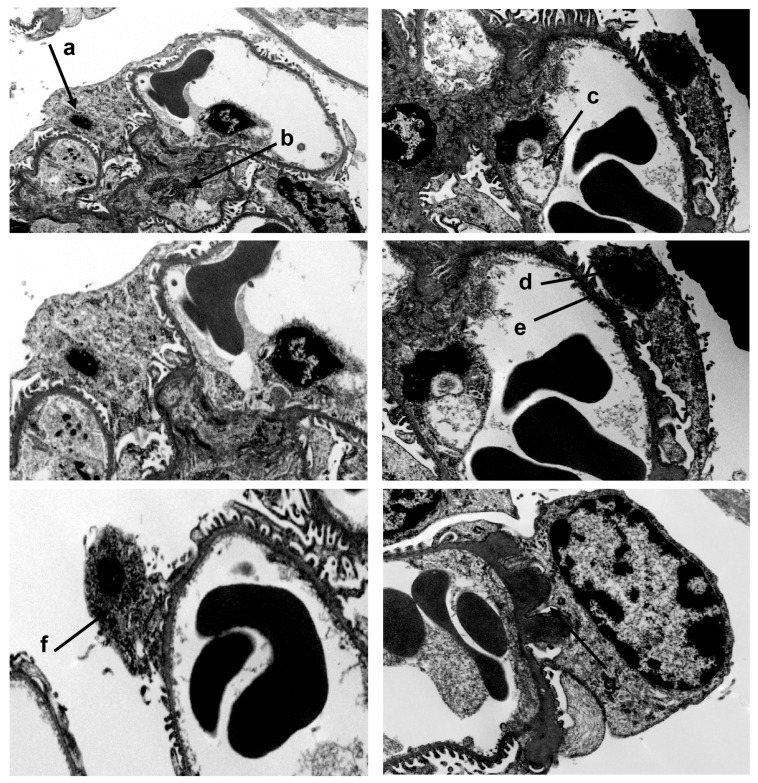
In 6-month-old A20Δpodocyte mice, we observed hypocellularity of the glomerular tuft, expansion of the extracellular matrix (b), podocytopenia associated with foot process effacement (e), nuclear chromatin condensation (d), micronuclei (a), and podocyte detachment at the ultrastructural level. In addition to podocyte death, we detected damage to intracapillary endothelial cells with vacuolation of the cytoplasm (c) and condensation of nuclear chromatin. Lower panel left: dying podocyte with micronuclei (f); lower panel right: irregular thickening of the GBM, looking like subepithelial deposit “humps” of immune complexes.

However, electron microscopy analyses of kidneys revealed significant alterations in the glomeruli of A20Δpodocyte mice. In 6-month-old A20Δpodocyte mice, we identified several ultrastructural changes, including glomerular tuft hypocellularity, expansion of the extracellular matrix, podocytopenia accompanied by foot process effacement, nuclear chromatin condensation, micronuclei, and podocyte detachment. In addition to podocyte death, we also detected impairment of intracapillary endothelial cells, characterized by cytoplasmic vacuolation and nuclear chromatin condensation (Figure 7). Additionally, A20Δpodocyte mice presented notable alterations, such as irregular glomerular basement membrane (GBM) thickening, resembling subepithelial deposits of immunocomplexes often referred to as “humps” (Figure 7, lower panel right). Moreover, the presence of dying podocytes with micronuclei was observed (Figure 7).

Collectively, these findings suggest that A20 in podocytes functions to safeguard against podocyte injury and subsequent loss.

### 3.4. Loss of A20 in Cultured Podocytes Results in Increased Cell Death and the Production of Chemotactic Mediators

**Figure 8:**
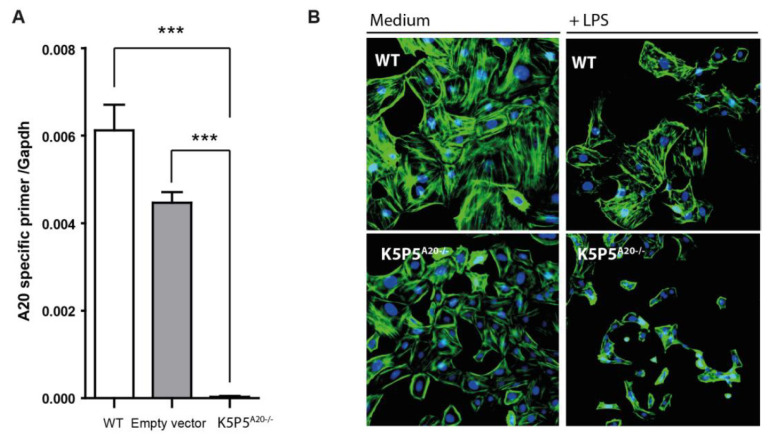
We generated a Tnfaip3 knockout model via the CRISPR/Cas9 system in podocytes. The specific gRNA was designed based on the DNA sequence of the mouse Tnfaip3 gene. This gRNA guides Cas9 to cut exon 1 of Tnfaip3. Single cells were selected via cell sorting (GFP+). (**A**) RT–PCR screening of single clones (*n* = 3) identified several K5P5^A20−/−^ clones. (**B**) Changes in F-actin and cell morphology after phalloidin staining of untreated and LPS-treated podocytes (24 h stimulation). Data are presented as mean ± SD. Statistical significance is indicated as follows: *** *p* < 0.001.

Next, we utilized CRISPR/Cas9 technology to obtain in vitro results. We anticipated that the complete absence of A20 expression achieved through CRISPR/Cas9 would result in more pronounced changes in cellular susceptibility to inflammation compared to those observed with siRNA technology (Figure 2). To elucidate the specific roles of *Tnfaip3* in podocytes, we coexpressed Cas9 with gRNA targeting exon 1 of the mouse *Tnfaip3* gene in the K5P5 podocyte cell line. Immunoblotting analysis verified the absence of the A20 protein in the knockout (KO) cell lines, K5P5^A20−/−^, but not in the wild-type (WT) or empty vector cell lines (Figure 8A). While the K5P5^A20−/−^ cell line exhibited normal growth, there was a slight alteration in phenotype during culture. Cellular stress-dependent actin cytoskeleton rearrangements are a well-known outcome of extensive inflammation [36,37].

We hypothesized that spontaneous and LPS-induced stress-dependent actin rearrangement could occur in K5P5^A20−/−^ podocytes. Thus, we stained WT or K5P5^A20−/−^ podocytes with FITC-conjugated phalloidin to visualize changes in actin organization following 24 h of LPS treatment and conducted a semiquantitative analysis of cytoskeletal alterations [38]. We observed differences in shape and cell attachment between WT and K5P5^A20−/−^ podocytes, reminiscent of some features of anoikis. K5P5^A20−/−^ podocytes exhibited increased cell rounding and detachment from neighboring cells, a phenomenon further exacerbated by LPS stimulation (Figure 8B).

**Figure 9:**
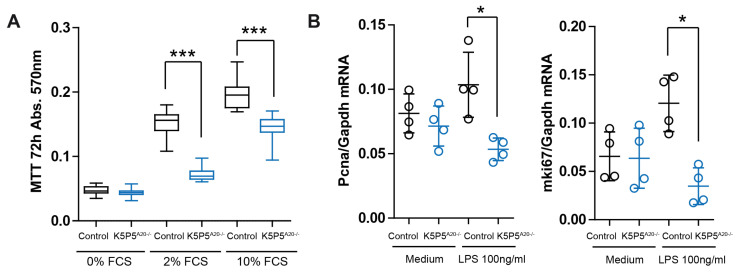
(**A**) Podocyte metabolic activity was assessed via the MTT assay (in 0–20% FCS). (**B**) Relative mRNA expression of *Pcna* associated with cell proliferation was characterized in 2% FCS. The data are shown as the means ± SDs. * *p* < 0.05 and *** *p* < 0.001.

Given the well-established significance of cell shape in determining cell fate, especially concerning cytoskeleton dynamics, which are crucial for podocyte structure and function [39,40], we conducted further experiments. The MTT assay, which characterizes cell metabolism, demonstrated significantly reduced tetrazolium salt (MTT), indicating lower cell metabolic activity in K5P5^A20−/−^ podocytes (Figure 9A). This reduction in metabolic activity may reflect decreased cell proliferation or increased cell death. We did not observe significant differences in the cell proliferation marker *Pcna* (Figure 9B).

**Figure 10:**
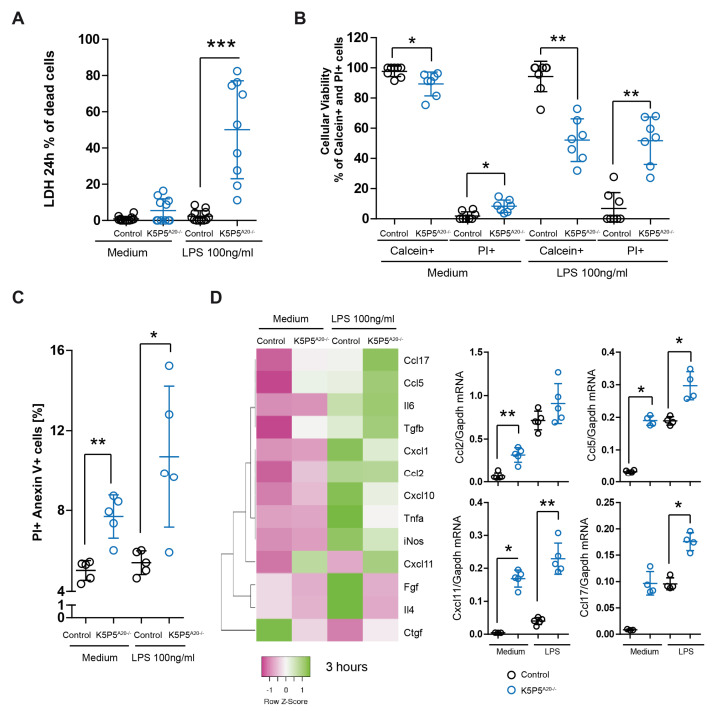
Podocytes were left untreated or exposed to LPS for 24 h, and viability was evaluated via (**A**) LDH assay and (**B**) fluorescence microscopy using PI staining and a calcein AM viability assay. (**C**) Podocytes were left untreated or exposed to LPS for 6 h, and viability was evaluated using PI and Annexin V flow cytometry analysis. (**D**) Heatmap of gene expression analysis of A20-deficient podocytes and controls that were left untreated or stimulated for 3 h with LPS. Quantitative real-time PCR analysis of selected transcripts in podocytes. The detected mRNA expression levels were calculated via the use of murine Gapdh as a housekeeping gene. The data are shown as the mean ± SDs. * *p* < 0.05, ** *p* < 0.01, and *** *p* < 0.001.

However, we detected significantly increased LDH levels in K5P5^A20−/−^ media following LPS stimulation (Figure 10A). Moreover, we employed fluorescent calcein and PI labeling to assess cell viability. Through fluorescence microscopy, we quantified viable calcein+ cells and cells with compromised cell membrane integrity (PI+ cells). A substantial increase in the percentage of dead cells was observed after 24 h of LPS incubation (Figure 10B), suggesting that the reduced metabolic activity in K5P5^A20−/−^ podocytes was primarily due to increased cell death. These results were confirmed by flow cytometry (Figure 10C). Moving on to chemokines, their dual functions as mediators of cell–microenvironment interactions, including chemotaxis and anoikis, present a plausible link to explain both the in vivo results (glomerular inflammation) and the knockout cell phenotype. Three hours after LPS stimulation, the expression of chemoattractants such as *Cxcl1*, *Cxcl10*, *Cxcl11*, *Ccl2*, *Ccl5*, and *Ccl17* was elevated in K5P5^A20−/−^ podocytes (Figure 10D). Chemokine genes were upregulated 2- to 50-fold in K5P5^A20−/−^ podocytes both upon LPS stimulation and in unstimulated conditions. Thus, we confirmed that A20 dysfunction in podocytes potentially contributes to increased podocyte death and promotes leukocyte recruitment into the glomerulus. Moreover, A20 in podocytes functions as a protective factor against glomerular inflammation by restraining chemokine production.

### 3.5. A20-Knockout K5P5^A20−/−^ Podocytes Are More Susceptible to Cytoskeleton Rearrangements and Show a Reduction in Integrin b1 Expression

A20 deficiency resulted in elevated chemokine levels and impaired adhesion and cell–cell interactions. Subsequent investigations focused on cell adhesion molecules and downstream components, specifically examining the gene expression of factors linked to inflammation, cell death, the cytoskeleton, and cellular dissociation following 18 h of LPS stimulation. Notably, K5P5^A20−/−^ podocytes exhibited sustained elevation of chemokine expression, along with dysregulation of cytoskeleton-related genes and cell death pathways, including anoikis. Several key genes implicated in anoikis, such as focal adhesion kinase (*FAK*) and *Rock2*, displayed significant alterations. Moreover, we observed diminished expression of transcripts encoding claudin-1, desmoplakin, and laminin in A20-deficient podocytes following LPS stimulation.

**Figure 11:**
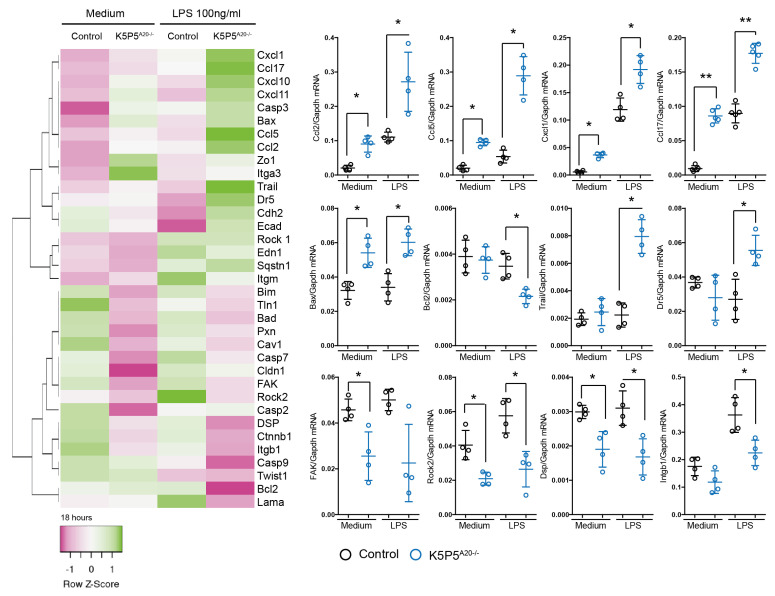
Heatmap depicting the expression patterns of cytoskeleton-related genes in A20-deficient podocytes and control podocytes, both untreated and stimulated with LPS for 18 h. Quantitative real-time PCR (qRT-PCR) analysis of selected transcripts in podocytes. mRNA expression levels were normalized to murine Gapdh, used as a housekeeping gene. Data are presented as mean ± SD. Statistical significance is indicated as follows: * *p* < 0.05 and ** *p* < 0.01.

**Figure 12:**
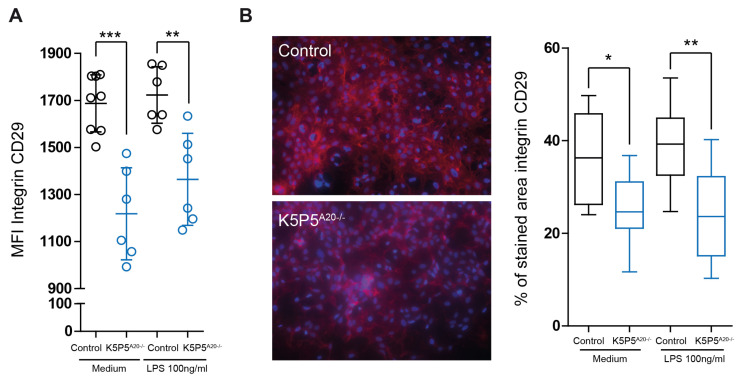
(**A**) Flow cytometric analysis of integrin. (**B**) Immunohistochemistry for integrin (*n* = 6). The data are shown as the means ± SDs. * *p* < 0.05, ** *p* < 0.01, and *** *p* < 0.001.

These proteins play crucial roles in maintaining cytoskeletal integrity. Furthermore, transcripts encoding paxillin, talin1, and caveolin-1, which are known to be involved in cytoskeleton-related cell death, were reduced both in knockout cells prior to stimulation and upon LPS stimulation (Figure 11). Given the robust datasets on anoikis and integrin β1, we proceeded to assess the expression of this protein on the cell surface via flow cytometry. Our findings revealed a significant reduction in the levels of integrin β1 on the cell surface (Figure 12A). This observation was further corroborated by immunohistochemistry (Figure 12B).

Collectively, these findings indicate that A20 exerts a comprehensive and substantial influence on cell adhesion proteins, the cytoskeleton, and matrix detachment-induced cell separation.

**Figure 13:**
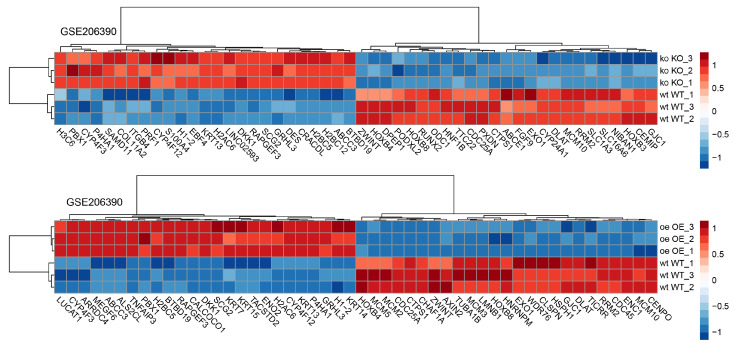
The heatmap displays the expression patterns of genes in HCT116 cells subjected to A20/Tnfaip overexpression (OE) and knockout (KO). Genes are arranged vertically, and the two conditions (OE and KO) are arranged horizontally for comparison; red: upregulated genes (high expression); blue: downregulated genes (low expression).

Our observations appear to apply to other cell types in which A20 is dysregulated, whether through overexpression (OE) or knockout (KO) [41]. The analyses of HCT116 cells demonstrated that many of the strongly regulated genes are involved not only in cell death but also in cytoskeletal rearrangements (Figure 13). Notably, genes regulating keratin intermediate filaments such as *KRT7*, *13*, *14*, and *15* were upregulated in A20 OE cells. Keratin networks play critical roles in adhesion, migration, and metabolism [42]. They are essential for maintaining cytoskeletal dynamics, enabling rapid and protein biosynthesis-independent remodeling of the network while preserving its integrity. Grainyhead-like transcription factor 3 (*Grhl3*) plays a crucial role as a downstream transcription factor in the Wnt/β-catenin pathway during epidermal differentiation. Studies have shown that Grhl3 promotes actomyosin network enrichment [43]. In A20 OE cells, *Grhl3* expression was similarly upregulated. Integrin β4 (*ITGB4*), which facilitates the bidirectional exchange of information between intracellular and extracellular matrices, has been implicated as a key regulator of tumor migration and invasion and was upregulated in A20 KO cells [44]. On the other hand, tubulin alpha 1b (*TUBA1B*), an important microtubule isoform involved in cytoskeleton formation, was found to be downregulated in A20 OE cells. Similar observations were made for ectodermal-neural cortex 1 (*ENC1*) and lamin B1 (*LMNB1*).

Collectively, these findings indicate that A20 exerts a comprehensive and substantial influence on cell cytoskeleton dynamics.

### 3.6. Gene Expression Analysis of TNFAIP3 and Selected Genes Involved in Anoikis, NF-κB Regulation, and Cell Attachment in Biopsies of Patients with Various Kidney Diseases

**Figure 14:**
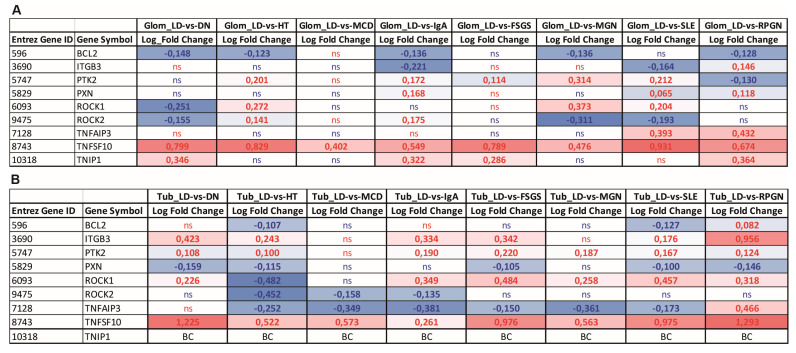
Gene expression analysis of *TNFAIP3* and selected genes involved in anoikis, NF-κB regulation, and cell attachment in the glomerular (**A**) and tubular (**B**) compartment of manually micro-dissected biopsies from patients with different kidney diseases. Values are expressed as log2-fold changes relative to controls (living donors/LDs). All displayed genes exhibit significant changes (*p* < 0.05), while non-significant genes are labeled as ns. Groups include Hypertensive Nephropathy/HT(N), Minimal Change Disease/MCD, IgA Nephritis/IgA, Rapidly Progressive Glomerulonephritis/RPGN, Systemic Lupus Erythematosus/SLE, Membranous Glomerulonephritis/MGN, Focal Segmental Glomerulosclerosis/FSGS, and Diabetic Nephropathy/DN. Red—upregulated genes, Blue—downregulated genes.

To assess the relevance of our cell and mouse model findings in human renal disease, we analyzed the transcriptional levels of *TNFAIP3* and selected genes involved in apoptosis and anoikis, NF-κB regulation, and cell attachment within both the glomerular and tubular compartments. Gene expression data from kidney biopsies of patients with various renal diseases (Figure 14), such as Diabetic Nephropathy (DN), Hypertensive Nephropathy (HT), Minimal Change Disease (MCD), IgA nephropathy (IgA), Focal Segmental Glomerulosclerosis (FSGS), membranous glomerulonephritis (MGN), Systemic Lupus Erythematosus (SLE), and rapidly progressive glomerunepritis (RPGN) were examined. In the glomerular compartment, an antiapoptotic gene involved in anoikis, *BCL2*, was downregulated in several diseases, though this change was not statistically significant in all cases. In contrast, the proapoptotic, anoikis-related gene *TNFSF10*, which encodes the TNF-related apoptosis-inducing ligand (TRAIL), was consistently upregulated across all disease groups, indicating a propensity for cell death, particularly in kidney diseases with strong inflammatory components. A20 inhibits TRAIL-mediated signaling and promotes *BCL2* expression, thus preventing excessive cell death and inflammation. This seems to be more prominent in tubular compartment, where downregulation of *TNFAIP3* and upregulation of *TNFSF10* were observed. Further, *TNFAIP3* is upregulated in the glomeruli of SLE and RPGN patients, while we did not observe expression dysregulation in glomeruli in other kidney diseases. TNIP1 [45] (TNFAIP3-interacting protein 1), exerting similar anti-inflammatory function as *TNFAIP3*, shows a trend toward upregulation in the glomerular compartment in human kidney diseases. Moreover, *ITGB3*, a gene known to participate in cell adhesion and multidirectional regulation of anoikis, displayed significant upregulation in the tubular compartment in most of the renal diseases and was downregulated in the glomeruli of patients with autoimmune diseases SLE and IgA. *PTK2/FAK1*, an anoikis resistance gene, was upregulated across all diseases in the tubular compartment as well as across a majority of the diseases in the glomerular compartment. Notably, *PXN*, a cytoskeleton-associated protein that suppresses anoikis, was downregulated in the tubular compartment. The tubular compartment expression pattern seem to be more consistent regarding the expression of anoikis-related genes. However, no consistent signature of anoikis resistance was identified across the glomerular diseases in the glomerular compartment.

In conclusion, these data show the high complexity and multidirectionality of the anoikis- and cell adhesion-associated gene regulation in human renal diseases.

## 4. Discussion

Various investigations have identified podocyte injury as a pivotal contributing factor in the development of glomerular injury [2,46,47,48]; however, the intricate molecular mechanisms governing podocyte homeostasis and its impact on the glomerular environment remain poorly understood. In this study, our primary aim was to explore the innate immune response of podocytes, with a focus on the role of Tnfaip3/A20. We hypothesized that (1) a podocyte-specific lack of A20 leads to a decline in kidney function and that (2) impaired A20 signaling in podocytes decreases the threshold for an inflammatory response but also influences the basal level of inflammation. Our in vivo data confirm that A20 is critical for podocyte maintenance of adequate kidney function, evidenced by the deterioration of kidney parameters in A20Δpodocyte mice. The functional decline was confirmed through the observation of changes in the glomerulus structure and a reduction in the number of podocytes. Moreover, both the knockdown and knockout of A20 increased the amount of chemokines that promote the chemotaxis of macrophages and neutrophils under basal conditions as well as upon stimulation with inflammatory mediators. These findings highlight the translational importance of understanding podocyte dysfunction. Podocyte injury, particularly through foot process effacement and ultrastructural damage, not only disrupts the filtration barrier but also creates a proinflammatory microenvironment that perpetuates glomerular disease. This underscores the need to consider podocyte health as a key therapeutic target in the treatment of kidney disorders. Our data support the well-established knowledge that A20 serves as a pivotal regulator of glomerular inflammation. The observed functional impairment and morphological alterations in the A20 podocyte-knockout group can be attributed to A20’s ability to inhibit NF-κB activation [49,50,51]. Mutations in the A20 gene have been implicated in a range of inflammatory disorders, including Crohn’s disease, Systemic Lupus Erythematosus, rheumatoid arthritis, and atherosclerosis [22,52,53,54,55]. Given the established role of A20 in these diseases and in regulating inflammation, it is plausible that similar mechanisms may be involved in renal pathologies. Indeed, A20 overexpression was shown to attenuate renal injury, improve histological features, and reduce cell apoptosis as well as NF-κB activation in a rat model of renal IRI [56]. Moreover, clinical investigations have revealed a correlation between podocyte loss and the progression of inflammatory glomerular diseases [46,57,58,59,60]. Recent studies conducted in mice revealed that A20 indirectly mitigates pathological inflammation by impeding various forms of cell death [61,62]. Priem et al. reported that A20 deficiency results in the simultaneous occurrence of RIPK1 kinase-dependent and -independent apoptosis but not in the spontaneous induction of necroptosis. Moreover, constitutively expressed A20 protects cells from TNF-induced death independently of its upregulated expression, indicating that two different pools of A20 regulate cell death and NF-κB activation [63]. Interestingly, another study revealed that TNFAIP3 restricts mTOR signaling and promotes autophagy, regulating survival in CD4 T cells [64]. Our results demonstrate that A20 protects podocytes from death while enhancing viability, suggesting that the regulation of innate immune responses and cell survival by A20 modulates podocyte injury and podocytopathies. From a clinical perspective, targeting pathways associated with podocyte survival, such as NF-κB signaling and A20-mediated protection, could pave the way for innovative treatments aimed at preserving podocyte integrity and preventing glomerular injury. Surprisingly, I325N A20 mutants, which exhibit increased NF-κB activation with inflammation, were protected in a kidney model of IRI. The I325N variant triggered an increase in NF-κB-dependent antiapoptotic factors, including BCL-2, BCL-XL, c-FLIP, and A20; reduced superoxide levels; and increased the activity of Foxp3+ T cells [65].

Our findings support the hypothesis that podocytes contribute to the inflammatory milieu in glomerular disease through cytokine/chemokine secretion [37,66]. Our data revealed that A20 is involved in the regulation of chemokine expression in podocytes and actively contributes to shaping the microenvironment within the kidney. The anticipated immunomodulatory role of A20 aligns with previous studies in this field, corroborating the existing body of evidence. For example, an in vitro examination revealed proinflammatory activation within ABIN1Δ podocytes [67]. Notably, ABIN1 inhibits NF-kB in an A20-dependent manner [68]. Consistent with prior research, Korte et al. demonstrated that supernatants from ABIN1-deficient podocytes accelerated the chemotaxis of human neutrophils and that these podocytes were susceptible to morphological alterations induced by neutrophil granule contents [67].

Moreover, our findings support the hypothesis that podocytes respond to the inflammatory milieu via rearrangement of the actin cytoskeleton, leading to effacement [37]. Our data revealed distinct growth patterns in A20-deficient cells compared with control podocytes. Furthermore, upon stimulation, we observed notable differences in cytoskeletal rearrangement, which indicated anoikis, a form of cell death associated with the detachment of cells from their extracellular matrix [69]. Anoikis, or the phenomenon of “lonely cell death”, might be crucial in podocyte biology because disruptions in the adhesion and connections between these cells can compromise the structural and functional integrity of the filtration barrier. The importance of an intact cytoskeleton is highlighted by the observation that genetic mutations affecting proteins related to podocyte morphology often underlie various glomerular diseases [70]. An intact podocyte cytoskeleton is a key determinant of filtration barrier integrity, and therapeutic strategies targeting cytoskeleton stabilization may help prevent the progression of glomerular diseases. Consequently, podocyte injury, including cytoskeleton alterations, induces foot process effacement (FPE), ultimately compromising the integrity of the filtration barrier [71,72,73,74]. This breach leads to the leakage of plasma proteins, including albumin, into the urine, which aligns with findings from our investigations [75]. Moreover, previous research has underscored the propensity of inflammation to induce alterations in the cytoskeletal framework [37,67,76,77]. In agreement with these prior findings, our results provide additional confirmation that severe inflammation can induce morphological alterations in podocytes, ultimately contributing to podocyte loss. Our results are in accordance with the widely accepted paradigm that both alterations in the cytoskeleton and the detachment of normally adhered cells are responsible for anoikis and that the mechanism behind the regulation of this process relies on integrin-mediated cell adhesion signals [78,79,80,81]. Notably, this type of cell phenotypical change followed by cell death has been observed in various cell lines, including epithelial cells [69,82], suggesting that a similar mechanism may operate in podocytes. We showed that key factors involved in anoikis, such as focal adhesion kinase [83], Rock2 [84], and integrin β1 [85], exhibited altered expression. Additionally, we observed a significant reduction in the expression of genes responsible for cell attachment and cytoskeleton reorganization, including desmoplakin, claudin-1, laminin, paxillin, talin1, and caveolin-1 [73,86,87,88]. Interestingly, in tumor models where A20 expression is increased, a correlation with reduced instances of anoikis was observed [89,90]. These findings raise the question of whether resistance to anoikis might bolster the capacity of A20-high tumors for metastasis. On the other hand, before undergoing anoikis, detached cells typically display morphological changes, such as adopting a rounded shape, contracting, rearranging their cytoskeleton, and experiencing nuclear alterations. In the context of metastasis, “lonely survival” may characterize individual cells’ ability to thrive in isolation and become detached from the primary tumor. Consequently, A20 has been reported to promote survival, potentially favoring the growth of cancer cells at metastatic sites; however, this process might be highly context-dependent. Our experiments were unable to pinpoint the precise mechanism by which A20 regulates cellular dissociation, leaving the nature of its role—whether direct or indirect—in this critical form of cell death open for future investigation. Interestingly, we did not observe a consistent signature of anoikis resistance across various glomerular diseases, suggesting that the role of A20 in regulating anoikis in podocytes is likely context-dependent and influenced by the interplay between glomerular and tubular compartments. This complexity may arise from the involvement of other cell types within the kidney microenvironment that contribute to the observed gene expression patterns. Furthermore, the progressive loss of podocytes and the increasing fibrosis seen in chronic and inflammatory renal conditions add another layer of difficulty in isolating A20’s specific role. Despite these challenges, the unique position of A20 as a modulator of both inflammation and cell survival highlights its potential as a therapeutic target. Strategies aimed at modulating A20 expression could offer dual benefits, such as mitigating inflammatory damage and preserving podocyte adhesion to the glomerular basement membrane, thereby preventing anoikis. Moreover, targeting A20 may have broader implications for reducing fibrosis and protecting overall glomerular architecture by stabilizing the microenvironment.

In summary, we propose that A20 maintains podocyte homeostasis and thereby contributes to podocyte response and cross talk with other cells within the glomeruli. By modulating inflammatory activity, cytoskeletal integrity, and cell survival, A20 plays a critical role in preserving kidney function. Understanding podocyte dysfunction is essential for developing interventions to treat and prevent various kidney diseases, including glomerular disorders and chronic kidney disease. Whether targeted modulation of A20 activity may allow the targeting of glomerular diseases is a question of translational relevance that needs to be addressed in the future.

## Data Availability

All data resulting from our study are presented within the paper. The datasets used for this study can be found in the Gene Expression Omnibus (GEO)—GSE99340, GSE32591, GSE35489, GSE47185, and GSE37463. We made use of online available RNA sequencing data (GSE184601: https://www.ncbi.nlm.nih.gov/gds/?term=GSE184601[Accession] (accessed on 22 September 2021) as well as GSE206390: https://www.ncbi.nlm.nih.gov/gds/?term=GSE206390[Accession] accessed on 17 June 2022) and included necessary information, such as dataset number and references, in the paper.

## Supplementary Materials

The following supporting information can be downloaded at https://www.mdpi.com/article/10.3390/cells14050381/s1: Table S1: List of primers used in the experiments, including their sequences and relevant details.
