# Supplementary material for: Podocyte A20/TNFAIP3 Controls Glomerulonephritis Severity via the Regulation of Inflammatory Responses and Effects on the Cytoskeleton
Source: Cells. 2025 Mar 5;14(5):381. doi: 10.3390/cells14050381 (PMC11898495; doi:10.3390/cells14050381)
Supplement: Supplementary file 1 [file cells-14-00381-s001.zip › cells-3394264-supplementary.pdf]

Table S1. List of primers used in the experiments, including their sequences and relevant details.

Official Symbol	NM number	FW (5' → 3')	RV (5' → 3')
HUMAN			
GAPDH	NM_002046	GTCTCCTCTGACTTCAACAGCG	ACCACCCTGTTGCTGTAGCCAA
TNFAIP 3	NM_001270507	GGACTTTGCGAAAGGATCG	TCACAGCTTTCCGCATATTG
MOUSE			
Bax	NM_007527	TTGCTGATGGCAACTTCAAC	GATCAGCTCGGGCACTTTAG
Bcl 2	NM_009741	GATCCAGGATAACGGAGGCT	GGTCTTCAGAGACAGCCAGG
Ccl 2	NM_011333	GCTACAAGAGGATCACCAGCA	GTCTGGACCCATTCCTTCTTG
Ccl 5	NM_013653	CCACTTCTTCTCTGGGTTGG	GTGCCCACGTCAAGGAGTAT
Ccl 17	NM_011332	TGCTTCTGGGGACTTTTCTG	ATAGGAATGGCCCCTTTGAA
Col 1a1	NM_007742	ACATGTTCAGCTTTGTGGACC	TAGGCCATTGTGTATGCAGC
Col 4a1	NM_009931	GTCTGGCTTCTGCTGCTCTT	CACATTTTCCACAGCCAGAG
Ctgf	NM_010217	AGCTGACCTGGAGGAAAACA	CCGCAGAACTTAGCCCTGTA
Cxcl 1	NM_008176	ACCCAAACCGAAGTCATAGCC	CTCCGTTACTTGGGGACACC
Cxcl 10	NM_021274	GGCTGGTCACCTTTCAGAAG	ATGGATGGACAGCAGAGAGC
Cxcl 11	NR_038116	CTGCTGAGATGAACAGGAAGG	CGCCCCTGTTTGAACATAAG
Dsp	NM_023842	TACACCTCAGGGCTGGAAACTC	GTAGTCTCCAGACCTCGTAAGC
Gapdh	NM_001289726	CATGGCCTTCCGTGTTCCTA	CCTGCTTCACCACCTTCTCA
Il 6	NM_031168	TGATGCACTTGCAGAAAACA	ACCAGAGGAAATTTTCAATAGGC
Itgb 1	NM_010578	CTCCAGAAGGTGGCTTTGATGC	GTGAAACCCAGCATCCGTGGAA
Mki 67	NM_001081117	GAAGTCAAAGAGCAAGAGGCAA	TCTTGAGGCTCGCCTTGATG
Nos 2	NM_010927	TTCTGTGCTGTCCCAGTGAG	TGAAGAAAACCCCTTGTGCT
Nphs 1	NM_019459	CACCTGTATGACGAGGTGGA	CAGCGAAGGTCATAAGGGTC
Nphs 2	NM_130456	TGGACGTGGACGAGGTTC	CCTAATCCAGAGGGCTTGAT
Pcna	NM_011045	TGGATAAAGAAGAGGAGGCG	GGAGACAGTGGAGTGGCTTT
Ptk 2	NM_001358045	ACATCAAGGCGTGTACCTGAGC	GTGAGGATGGTCAAACTGACGC
Synpo	NM_177340	GCCAGGGACCAGCCAGATA	AGGAGCCCAGGCCTTCTCT
Tgfb 1	NM_011577	GGAGAGCCCTGGATACCAAC	CAACCCAGGTCCTTCCTAAA
Tjp 1 (ZO1)	NM_009386	GTTGGTACGGTGCCCTGAAAGA	GCTGACAGGTAGGACAGACGAT
Tnf alpha	NM_013693	AGGGTCTGGGCCATAGAACT	CCACCACGCTCTTCTGTCTAC
Tnfaip 3 (A20)	NM_009397	AAGCTCGTGGCTCTGAAAAC	TTCCTCAGGACCAGGTCAGT
Tnfrsf 10b (Dr5)	NM_020275	TGTGTCGATGCAAACCAGGCAC	GCCGTTTTGGAGACACACTTCC
Tnfsf 10 (TRAIL)	NM_009425	GGAAGACCTCAGAAAGTGGCAG	TTTCCGAGAGGACTCCCAGGAT
Vegfa	NM_001287057	CTGCTGTAACGATGAAGCCCTG	GCTGTAGGAAGCTCATCTCTCC
Wt 1	NM_144783	CCAGCTCAGTGAAATGGACA	CTGTACTGGGCACCACAGAG
